# Health-Related Quality of Life in the Era of Immune Checkpoint Blockade: What Do Patient-Reported Outcomes Reveal?

**DOI:** 10.3390/cancers17243917

**Published:** 2025-12-07

**Authors:** Alexandra M. Dunker, Neha Malik, Kathryn J. Krause, Emily Z. Keung, Jason B. Liu, Elise F. Nassif Haddad, Neeta Somaiah, Heather G. Lyu, Christina L. Roland

**Affiliations:** 1 Department of Surgical Oncology, The University of Texas MD Anderson Cancer Center, Houston, TX 77030, USA; amdunker@mdanderson.org (A.M.D.);; 2 Research Medical Library, The University of Texas MD Anderson Cancer Center, Houston, TX 77030, USA; 3 Department of Sarcoma Medical Oncology, The University of Texas MD Anderson Cancer Center, Houston, TX 77030, USA

**Keywords:** immunotherapy, cancer survivorship, immuno-oncology, health utility, symptom burden

## Abstract

Background: Immune checkpoint inhibitors (ICIs) have revolutionized cancer treatment by providing durable survival gains, but understanding their effects on patient health-related quality of life (HRQL) is critical. Methods: We performed a narrative review of cross-sectional surveys, early-phase trials, and large-scale phase II and III randomized controlled clinical trials assessing FDA-approved ICIs, including programmed cell death protein 1 (PD-1) inhibitors, programmed death ligand 1 (PD-L1) inhibitors, and cytotoxic T lymphocyte-associated antigen 4 (CTLA-4) inhibitors, with emphasis on patient-reported HRQL. Validated HRQL instruments were summarized, and for pivotal trials, the positioning of HRQL outcomes as primary, secondary, or exploratory endpoints was taken from original protocols or primary manuscripts. Results: ICIs generally preserved or improved HRQL in patients with various malignancies compared with chemotherapy, targeted therapies, or observation. PD-1/PD-L1 inhibitors maintained global health and function and delayed symptom progression in patients with lung cancer, melanoma, and renal cell carcinoma. Regimens combining CTLA-4 blockade and PD-1/PD-L1 inhibition (e.g., nivolumab + ipilimumab, durvalumab + tremelimumab) are associated with HRQL outcomes similar or superior to those of targeted therapies. Overall, most immune-related adverse effects were short-term and did not diminish HRQL benefits. Conclusions: ICIs extend survival while preserving, and often enhancing, patient HRQL. These medications represent a shift in oncology, offering not just longer life but also better daily well-being. Continued long-term patient-reported outcome monitoring is essential to guide survivorship care in the immunotherapy era.

## Simple Summary

Immune checkpoint inhibitors are treatments that work by strengthening the body’s immune response against tumors. This narrative review summarizes patient-reported data on how these medications affect the well-being of patients with cancer, using quality-of-life questionnaires that have been validated and used in many trials and observational studies. Overall, treatments that target PD-1, PD-L1, or CTLA-4 preserve or even improve the physical, emotional, and social functioning of patients with cancer compared with chemotherapy or observation. There is, however, a risk of immune-related adverse effects that are manageable. Improving routine standardized collection and long-term follow-up of these patient-reported outcomes will lead to better-informed treatment decisions and survivorship care, enabling patients to have a longer life with better health quality.

## 1. Introduction

In recent years, immunotherapy has transformed the therapeutic landscape for many malignancies, offering durable responses and survival benefits in patient populations historically limited to palliative or short-term interventions [1,2]. Among the most widely used agents are immune checkpoint inhibitors (ICIs), which function by blocking inhibitory pathways that limit T-cell activation. These include programmed cell death protein 1 (PD-1) inhibitors (nivolumab, pembrolizumab, cemiplimab, dostarlimab, toripalimab, tislelizumab, camrelizumab, and sintilimab), programmed death ligand 1 (PD-L1) inhibitors (atezolizumab, durvalumab, avelumab, envafolimab, and cosibelimab), and cytotoxic T lymphocyte-associated antigen 4 (CTLA-4) inhibitors (ipilimumab and tremelimumab) [3]. By enhancing antitumor immune activity, these agents have produced significant improvements in outcomes across a range of cancers, including melanoma, non-small cell lung cancer (NSCLC), and renal cell carcinoma [4,5,6].

While survival outcomes are central to cancer care, the effect of treatment on health-related quality of life (HRQL) is an equally critical consideration [7]. Patients receiving immunotherapy often experience distinct toxicity profiles with immune-related adverse events ranging from dermatologic and endocrine disturbances to severe colitis, pneumonitis, and hepatitis [3]. These adverse effects, alongside the potential for long-term disease control, can shape patients’ overall well-being, functioning, and daily life in ways that differ substantially from experiences with conventional systemic therapies [8,9].

To assess these multidimensional outcomes, several validated patient-reported outcome (PRO) instruments have been applied in immunotherapy studies. Commonly used scales include the Functional Assessment of Cancer Therapy (FACT) [10], the MD Anderson Symptom Inventory (MDASI) [11], the Patient-Reported Outcomes Measurement Information System (PROMIS) [12], the European Organisation for Research and Treatment of Cancer Quality of Life Questionnaire (EORTC QLQ-C30) [13], and the EuroQol 5-Dimension instrument (EQ-5D) [14]. These tools capture domains such as physical, emotional, and social functioning, allowing for a comprehensive evaluation of the impact of immunotherapy beyond clinical endpoints [7,15] (Table 1).

Given the rapid expansion of immunotherapy indications and the heterogeneity of available data, a systematic synthesis of how different ICIs influence patient-reported HRQL is warranted. This review summarizes the current evidence on HRQL outcomes across major immunotherapy classes, highlighting both common trends and agent-specific considerations. To this end, we conducted an extensive literature search in Ovid Medline and Ovid Embase using a wide variety of MeSH and EMTREE subject headings and keywords such as “quality of life” and “symptom burden” combined with drug terms and a variety of other concepts such as “patient reported outcomes,” “surveys and questionnaires,” “measurement tools,” and specific tools such as EORTC. Searches were not restricted by date or study type. We then reviewed cross-sectional surveys, early-phase trials, and large-scale phase II and II clinical trials that reported HRQL measures in cancer patients receiving PD-1, PD-L1, or CTLA-4 inhibitors. Recent survivorship research reinforces the importance of this approach. Cross-sectional studies in long-term ICI-treated melanoma and mixed-cancer populations demonstrate that while global HRQL often remains high, a substantial subset of survivors continue to experience persistent fatigue, emotional distress, or musculoskeletal symptoms 1–5 years after therapy, underscoring the need for systematic long-term PRO assessment [16,17].

## 2. PD-1 Inhibitors

ICIs targeting PD-1 (nivolumab, pembrolizumab, cemiplimab, and others) have demonstrated not only survival benefits but also favorable PROs in many cancers compared with conventional therapies. In advanced NSCLC, multiple trials have shown that PD-1 inhibitors are associated with maintenance of or even improvements in HRQL compared with chemotherapy. For example, in the CheckMate 057 study (nivolumab vs. docetaxel for progressing NSCLC), patients on nivolumab reported better lung cancer symptom control and overall health status over time than those treated with docetaxel [9]. Similarly, first-line pembrolizumab led to improved or preserved HRQL in PD-L1–positive NSCLC: in KEYNOTE-024, pembrolizumab-treated patients had stable or improved global HRQL scores at 3–4 months while chemotherapy patients experienced a decline [4]. Pembrolizumab also significantly delayed time to deterioration of HRQL (for symptoms such as cough, pain, and dyspnea) relative to chemotherapy [4], reflecting the gentler toxicity profile of immunotherapy. Newer anti–PD-1 agents introduced in recent years seem to follow the same trend, as patients with NSCLC with brain metastases experienced improved cognitive function and HRQL scores over time during treatment with camrelizumab (PD-1 inhibitor) plus chemotherapy [18]. These findings indicate that PD-1 blockade, by avoiding or reducing the cumulative toxicities of cytotoxic drugs, often allows patients to feel better or no worse than they do on cytotoxic treatments while their cancer is controlled.

Beyond lung cancer, similar HRQL benefits of PD-1 inhibitors have been observed in other malignancies. In patients with previously treated advanced renal cell carcinoma, nivolumab monotherapy not only improved survival versus everolimus but also yielded better patient-reported well-being. A post hoc analysis of CheckMate 025 showed significantly greater improvements versus baseline assessments on the Functional Assessment of Cancer Therapy Kidney Symptom Index (FKSI-DRS) with nivolumab; more than half of nivolumab-treated patients had clinically meaningful symptom relief, versus only 37% with everolimus [19]. Patients on nivolumab also achieved this HRQL improvement faster (median ~4.7 months) than those on everolimus [19]. These results underscore that immunotherapy can enhance daily functioning in renal cell carcinoma patients by mitigating symptoms (fatigue, appetite loss, etc.) that often accompany targeted therapy.

In advanced metastatic melanoma, single-agent PD-1 inhibitors have likewise shown favorable HRQL profiles. In the CheckMate 067 trial, nivolumab monotherapy maintained a stable global health status in patients with advanced melanoma, with no significant deterioration over time compared with ipilimumab (CTLA-4 inhibitor), which has also been shown to maintain a stable HRQL in this population [6]. This stable HRQL profile for nivolumab aligns with clinical experience in patients with advanced melanoma that PD-1 inhibitors cause fewer severe adverse effects than CTLA-4 blockade, allowing patients to carry on with normal activities to a greater extent [20]. Taken together, evidence across cancer types suggests that PD-1 inhibitors generally preserve or enhance HRQL relative to older standard-of-care chemotherapy, presenting with fewer of the debilitating day-to-day effects, yet still achieving durable clinical responses.

In addition to clinical trial data, long-term observational studies now shed light on HRQL beyond active treatment. A survey of advanced melanoma survivors treated with anti-PD-1 therapy found that although overall HRQL was generally good, approximately one-third reported ongoing fatigue or limitations in physical and emotional functioning several years after treatment completion [16]. Similarly, in a mixed-tumor survivorship cohort more than two years after starting PD-1/PD-L1 therapy, global HRQL scores were approximately equal to population norms, but neurocognitive symptoms, anxiety, depression, and sexual health concerns were still prevalent in a meaningful minority of patients [17]. These studies show that the benefits of PD-1 therapy often extend well into survivorship while also revealing symptoms remain that warrant ongoing assessment.

## 3. PD-L1 Inhibitors

Agents targeting the PD-L1 ligand (atezolizumab, durvalumab, avelumab, envafolimab, cosibelimab, etc.) have produced HRQL outcomes similar to those of PD-1 blockers, frequently showing no HRQL detriment and sometimes improvement when used instead of or alongside conventional therapy. In first-line treatment of NSCLC, for example, atezolizumab monotherapy was associated with better patient functioning and stable symptom control relative to platinum-doublet chemotherapy. Patients receiving atezolizumab in the IMpower133 trial had numerically improved physical functioning scores over time and no worsening of lung cancer–related symptoms, whereas chemotherapy patients saw greater symptom burden over time [21]. Likewise, in extensive-stage small-cell lung cancer, adding atezolizumab to standard carboplatin-etoposide not only improved survival but also produced more pronounced and persistent HRQL improvements than chemotherapy alone [21]. Both arms reported early HRQL gains (likely because tumors responded to treatment), but the atezolizumab arm’s gains were more sustained, highlighting that immunotherapy did not introduce new detriments to patients’ well-being and may have extended the period of symptom relief.

PD-L1 inhibitors have also been studied in settings where the alternative was no active therapy or maintenance therapy only, and findings consistently show no compromise in HRQL despite prolonged treatment. The PACIFIC trial in stage III NSCLC, which gave 1 year of durvalumab consolidation versus placebo after chemoradiotherapy, is illustrative. Over 12 months of therapy, patients on durvalumab reported similarly stable symptoms and functioning (assessed by EORTC QLQ-C30 questionnaires) compared with those receiving placebo/observation [22]. Importantly, there were no clinically significant between-group differences in key domains such as cough, dyspnea, fatigue, or global health status [22]. In other words, the survival benefit of durvalumab in this curative-intent setting came without degrading patients’ HRQL—a crucial finding given that the control arm had essentially no treatment and was expected to maintain HRQL. Complementing these findings, recent perioperative immunotherapy data from early-stage NSCLC show preserved HRQL. In the KEYNOTE-671 trial, adding pembrolizumab to neoadjuvant chemotherapy and adjuvant therapy did not compromise global health status or physical functioning compared to chemotherapy alone, indicating that HRQL can be maintained even in the curative-intent multimodal regimens [23]. Another notable example is avelumab in advanced bladder cancer. In the JAVELIN Bladder 100 trial, switch-maintenance therapy with avelumab after chemotherapy significantly extended overall survival in urothelial carcinoma, yet PROs (using FACT-Bladder and EQ-5D instruments) remained similar to those seen with best supportive care alone. The addition of avelumab did not lead to any significant deterioration in HRQL or symptoms; time to HRQL deterioration was similar between the avelumab plus supportive care versus supportive care–only groups [24]. Patients thus derived a survival advantage from immunotherapy without sacrificing their daily well-being, reinforcing the tolerability of PD-L1 blockade. Notably, published HRQL data for some newer PD-L1 agents (such as subcutaneously delivered envafolimab or the investigational cosibelimab) are still limited. Overall, the class effect for PD-L1 inhibitors appears to be that they substantially preserve baseline HRQL. Across trials in lung, bladder, and other cancers, patients on anti–PD-L1 therapy generally reported stable or improved functioning and symptom control, even as these treatments extended survival beyond what prior standards achieved [24]. This alignment of clinical efficacy with maintained HRQL underscores a major advantage of immunotherapy in oncology practice.

## 4. CTLA-4 Inhibitors and Combination Immunotherapy

CTLA-4-blocking antibodies (ipilimumab and tremelimumab) have a different toxicity profile from PD-1/PD-L1 inhibitors that raised concerns about HRQL in the early immunotherapy era. Ipilimumab monotherapy can result in significant immune-related effects (e.g., colitis, dermatitis, endocrinopathies), which might be expected to impair patient well-being during active treatment. Indeed, some patients on ipilimumab experience transient drops in HRQL when moderate to severe immune toxicities occur. However, clinical trials suggest that long-term HRQL is not substantially worse with CTLA-4 inhibitors, especially when they are combined with PD-1/PD-L1 agents for greater efficacy.

In advanced melanoma, the combination of nivolumab plus ipilimumab produced more frequent high-grade toxicities than ipilimumab alone, yet reported HRQL outcomes did not significantly differ between the arms. The CheckMate 067 study, a phase III trial assessing nivolumab plus ipilimumab efficacy in advanced melanoma, found that patients receiving nivolumab alone or with ipilimumab maintained a stable global health status over time, with no clinically meaningful deterioration compared with those on ipilimumab monotherapy [6]. Thus, despite added adverse effects, the nivolumab-containing regimens did not translate into worse overall HRQL. Many patients who had to discontinue treatment due to adverse events still reported rebound or stable HRQL afterward, likely because immune toxicities are often reversible with management and because the disease control from treatment can improve symptoms (e.g., tumor pain, fatigue) in the long run. This finding—that more toxicity does not always equal worse patient-reported HRQL—is encouraging, suggesting effective management of immune adverse effects can mitigate their impact on daily life.

When CTLA-4 inhibitors are used in combination regimens, outcomes generally mirror what is seen with PD-1/PD-L1 therapies alone or even show HRQL gains over previous standards. In first-line treatment of renal cell carcinoma, for instance, nivolumab plus ipilimumab demonstrated superior survival to sunitinib (a tyrosine kinase inhibitor) and also significantly better HRQL scores. Patients on the immunotherapy doublet reported higher FKSI-19 (kidney cancer HRQL index) scores than those on sunitinib, indicating fewer symptoms and better well-being, and this outcome was consistent at every point of follow-up through 2 years [25]. Many chronic toxicities of VEGF-targeted therapy (hand-foot syndrome, diarrhea, etc.) were avoided, and patients on nivolumab plus ipilimumab had longer intervals without disease symptoms or treatment adverse effects over the course of therapy [25]. Likewise, the durvalumab plus tremelimumab regimen in unresectable hepatocellular carcinoma improved survival versus the kinase inhibitor sorafenib while providing a more favorable patient experience. In the phase III HIMALAYA trial, both the immunotherapy combination (STRIDE regimen: single tremelimumab dose + durvalumab) and durvalumab alone yielded slower time to HRQL deterioration and higher odds of HRQL improvement than sorafenib [26]. The median time to degradation of global health status was about 7.5 months with STRIDE (7.4 months with durvalumab) versus only 5.7 months with sorafenib [26]. Patients on immunotherapy were more likely to report improvements in functioning and cancer-related symptoms during treatment, whereas those on sorafenib experienced earlier worsening in domains such as physical functioning, fatigue, appetite loss, and pain [26]. Notably, combining tremelimumab with durvalumab did not impair HRQL compared with durvalumab alone–both arms were similar on patient-reported indices, despite the combination’s extra initial toxicity [26].

Treatment sequence may also influence long-term patient experience. In stage III melanoma, a cross-sectional comparison showed that patients receiving neoadjuvant ICI had significantly better long-term HRQL, including higher physical, cognitive, and social functioning and lower fatigue, than those treated with adjuvant ICI regimens, suggesting that shorter-course neoadjuvant immunotherapy may confer a more favorable survivorship profile [27]. These findings indicate that treatment sequence and duration may be important considerations when interpreting HRQL outcomes associated with combination regimens.

In summary, although treatment with anti-CTLA4 therapies is often associated with increased toxicity, treatment can be delivered without causing unnecessary long-term HRQL burdens. Patients must be monitored and managed for immune-mediated adverse effects, which can temporarily affect HRQL. However, the evidence indicates that effective immunotherapy often leads to better disease symptom control, longer periods of high functioning, and similar or improved overall HRQL relative to previous standards treatments like chemotherapy or tyrosine kinase inhibitors [25,26]. The integration of CTLA-4 blockade into treatment (e.g., ipilimumab added to nivolumab, or tremelimumab added to PD-L1 therapy) has not eroded patient-reported well-being in trials—a testament to the adaptability of patients and clinicians in managing adverse effects, and to the benefit of achieving tumor regression that can alleviate cancer-related symptoms. HRQL outcomes associated with each ICI regimen discussed are summarized in Table 2.

## 5. Common Themes in HRQL with Immunotherapy Use

Across the different classes of ICIs, a clear trend emerges: patients’ HRQL is better preserved while on immunotherapy than the traditional cytotoxic therapies. Whether in metastatic melanoma, lung cancer, kidney cancer, or others, treatment with PD-1/PD-L1 or CTLA-4 inhibitors often results in stable or improved PROs when compared head-to-head with chemotherapy, targeted drugs, or placebo observation. Several common factors likely contribute to this favorable HRQL profile. First, ICIs have toxicity profiles that, while unique, generally lack the consistent adverse effects of cytotoxic chemotherapy (such as severe cytopenias, neuropathy, or universal hair loss). Many patients on immunotherapy report feeling more capable of maintaining daily activities, with less fatigue and physical decline than one might expect on intensive chemotherapy regimens [19,21]. Second, by producing durable tumor responses and prolonging survival, immunotherapies can relieve cancer-related symptoms (pain, cough, dyspnea, etc.) over the long term, supporting better functioning. Trials like CheckMate 057, KEYNOTE-024/042, and HIMALAYA all demonstrated that as tumors shrank or stabilized, patients experienced meaningful improvements in symptoms and global health that were sustained longer than in comparator arms [19,26]. Third, while immune-related adverse events do occur and can be serious, they are generally reversible with proper management and tend to be limited to specific organ systems rather than causing a generalized decline in health. For example, a patient might temporarily struggle with colitis or pneumonitis from ICI therapy, but with corticosteroids and care, these events often resolve; survivors can then return to a normal or near-normal HRQL, especially if their cancer is responding. This is reflected in studies (e.g., CheckMate 067, in melanoma) where even patients who discontinued therapy owing to toxicity could report rebound in HRQL scores during follow-up [6]. Long-term survivorship data echo these patterns. Studies of melanoma and multi-cancer ICI survivors report that, even years after treatment, most patients maintain good overall HRQL, though persistent fatigue, joint symptoms, neurocognitive difficulties, and mood disturbances remain for a subset [16,17]. These real-world observations support clinical trial evidence showing that while ICIs rarely cause broad functional decline, chronic low-grade symptoms may influence day-to-day well-being long after treatment.

It is also important to acknowledge that quality of life is multidimensional, and different PRO instruments have been used across these trials to capture it. These include general cancer HRQL questionnaires like the EORTC QLQ-C30 (with disease-specific modules such as QLQ-LC13 for lung cancer [21] or QLQ-HCC18 for liver cancer [26], symptom indices like the Lung Cancer Symptom Scale and FKSI for kidney cancer, and utility measures like EQ-5D. Despite the variety of tools, the overall narrative is consistent: ICI therapies enable patients to live better with their cancer. In practical terms, this means patients on immunotherapy often remain active longer, experience fewer daily symptoms, and require fewer palliative interventions than those on prior standards of care [9,25]. As patient-reported data are increasingly integrated into oncology trials, it is reassuring to clinicians and patients alike that ICI-based regimens generally demonstrate improvements in survival without coming at the cost of patients’ HRQL, fulfilling the dual goals of cancer treatment.

Moving forward, continued collection of HRQL data in immunotherapy studies—including longer-term survivorship follow-up—will be crucial. Early evidence already suggests that a subset of immunotherapy-treated patients enjoy years of disease control with good functional status, raising new questions about rehabilitation, return to work, and other survivorship aspects that were less relevant in the era of short-term metastatic cancer outcomes. By synthesizing the data to date, we see that ICIs, used appropriately, allow patients not just to live longer but also to live better during those added months and years [24,26]. This represents a paradigm shift in oncology, aligning treatment success with the patient’s lived experience, and underscores why immunotherapies have been so transformative in modern cancer care.

## 6. Gaps in the Literature and Future Directions

Despite the transformative survival gains from ICIs, patient-reported HRQL outcomes in immunotherapy trials remain inconsistently measured and reported. A recent systematic review of trials leading to immunotherapy approvals found that PRO data appeared in only ~48% of trial publications, often as secondary analyses without predefined hypotheses. Most studies use generic HRQL instruments (e.g., EORTC QLQ-C30 or EQ-5D) [30], yet these may underrepresent the unique toxicity profile of ICIs. In fact, Faury et al. note that standard HRQL tools “may fail to capture important symptomatology unique to ICIs” [30], and none have high quality evidence that have validated them for use in cancer patients for immunotherapy effects [30]. Moreover, trials typically publish PRO results in companion papers (often 1–2 years after primary efficacy results) [30], and very few prespecify PRO hypotheses or robust analysis plans [30]. This methodological heterogeneity (in instrument choice, timing of assessments, and analytic rigor) harms cross-trial comparison and synthesis of HRQL outcomes.

These inconsistencies have left critical data gaps for certain drugs and populations. For example, none of the pivotal ICI trials for the newer PD-L1 inhibitors durvalumab or avelumab [30] reported HRQL, likely reflecting delays or omissions in dedicated PRO publications. Similarly, complex regimens (e.g., dual ICI combinations, chemo–immunotherapy in bladder cancer or hepatocellular carcinoma) often lack published patient-reported outcomes altogether. Trial cohorts are also skewed: although many studies enroll patients internationally, few analyze HRQL by race, age, ethnicity or geography. Underrepresented minority groups and regions (e.g., rural areas, low-income countries) are rarely separately assessed, raising questions about generalizability. In practice, most HRQL data come from Western-centric randomized controlled trials, and post-marketing or real-world studies rarely incorporate longitudinal PRO collection. As a result, the lived experience of diverse patient groups on immunotherapy remains understudied. Even in trials that report PROs, the scope is often limited predominantly to symptom burden. Domains such as physical function, fatigue impact, emotional distress, and ability to maintain social and occupational roles, each highly relevant to long-term cancer survivorship, are inconsistently assessed or not included at all.

A particularly striking gap is the lack of long-term survivorship HRQL data. As patients live longer on ICIs, chronic symptoms may persist well beyond trial endpoints, but follow-up in most trials stops at 1–2 years. Only a handful of cross-sectional studies (almost exclusively in melanoma) have examined late effects. For instance, one survey of melanoma survivors (median ~2 years off therapy) found that 30% still reported fatigue and impaired physical/emotional function [16]. Another long-term melanoma cohort (1–5 years post-ICI) reported persistent fatigue (28%), pain, and anxiety/depression in substantial minorities of patients, despite generally good overall HRQL [31]. These data underscore that chronic immune-related adverse events (fatigue, arthralgias, neurocognitive effects, endocrinopathies) can erode HRQL in surviving patients. Crucially, analogous survivorship studies are missing in other tumors (NSCLC, renal cell carcinoma, bladder cancer, hepatocellular carcinoma), so the long-term impact of immunotherapy in these populations is unknown. This lack of extended follow-up and real-world HRQL research is a major literature gap that future cohorts or registry-based studies should address. Emerging survivorship studies provide insight into what such long-term evaluations might reveal. For example, Looman et al. [16] reported that nearly one-third of advanced melanoma survivors previously treated with anti-PD-1 therapy experienced ongoing fatigue or emotional/physical impairments several years after treatment cessation. Similarly, a large cross-sectional analysis across melanoma, NSCLC, urothelial, and renal cell carcinoma survivors found that although mean HRQL exceeded clinically relevant thresholds, 28% still exhibited lower global QOL, and notable subsets reported neurocognitive impairment, depression/anxiety, or sexual dysfunction more than two years after initiating ICIs [17]. These findings highlight that chronic immune-related effects–neurocognitive, endocrine, musculoskeletal, and psychosocial—may persist well beyond trial follow-up windows. HRQL may also vary by treatment timing and strategy. In stage III melanoma survivors, long-term HRQL was significantly better with neoadjuvant ICI than with adjuvant ICI, suggesting that regimen design, including duration and sequencing, may influence survivorship quality [27]. This underscores the need for future trials to capture HRQL not only during therapy but also years after treatment has ended.

To fill these gaps, future research must standardize and deepen HRQL assessment in ICI therapy. Consensus guidelines (e.g., 2009 FDA guidance, SISAQOL, or Setting International Standards in Analysing Patient-Reported Outcomes and Quality of Life Endpoints) should be applied so that trials prospectively define PRO endpoints, sampling schedules, and handling of missing data. PRO instruments may need augmentation or redesign. Since generic tools omit many immune-related adverse event domains, studies could incorporate targeted item libraries (for example, selected PRO-Common Terminology for Adverse Events questions for immune toxicities) or develop ICI-specific modules [30]. Importantly, HRQL endpoints should be integrated (even as co-primary outcomes) in trials of new agents and combinations, rather than added only post hoc. Digital health technologies offer promising avenues for continuous monitoring: for example, one Chinese randomized trial showed that weekly electronic PRO symptom monitoring with alert algorithms reduced serious immune-related adverse events, reduced ER visits, and preserved patients’ global HRQL [32]. Similarly, current protocols (e.g., the *BMJ Open* PRO-NET study) are testing smartphone-based symptom tracking with automated feedback for NSCLC patients on immunotherapy [33]. Embedding such ePRO systems into practice could capture real-time HRQL changes across therapy cycles and into survivorship. Equally, deliberate efforts should be made to enroll and report on diverse populations, so that HRQL findings reflect all patient groups. In summary, closing these gaps will require harmonized PRO methodologies, innovative monitoring tools, and dedicated survivorship cohorts, ensuring that the patient’s voice guides the next era of immuno-oncology research [32].

## 7. Conclusions

ICIs have reshaped cancer treatment as a field, helping patients not only live longer but also live better. The studies and cancer types in this review have shown that treatments targeting PD-1, PD-L1, and CTLA-4 have generally maintained or even improved patients’ HRQL compared with older standards like chemotherapy or targeted drugs. Even when immune-related adverse effects occur, they are generally manageable and often offset as ICIs help relieve cancer-related symptoms and lead to the recovery of normal functioning for patients. These results show that modern immunotherapy offers patients more than survival, supplying time that feels meaningful and is not as painful.

Nonetheless, survivorship studies also make clear that a minority of patients continue to experience fatigue, emotional distress, neurocognitive concerns, or sexual health problems long after treatment, emphasizing the importance of ongoing monitoring and more nuanced HRQL tools that capture these late effects [16,17]. Developing better tools to capture the effects of immunotherapy and using digital methods to track symptoms in real time will allow us to better understand how patients are experiencing these treatments. As immunotherapy increasingly cures or chronically controls cancer, long-term HRQL will remain central to evaluating its full impact on patients’ lives. Ultimately, aligning treatment success with how patients feel and function should remain at the heart of cancer care in the immunotherapy era.

## Figures and Tables

**Table 1:** Commonly Used HRQL Instruments in Immunotherapy Studies.

Measure	Primary Purpose	Main Domains and Subscales Assessed	Population and Setting
FACT [10]	Evaluates multidimensional quality of life in cancer patients	Physical well-being Social/family well-being Emotional well-being Functional well-being Disease-specific subscales (e.g., FACT-L for lung cancer)	Cancer patients across different cancer types
MDASI [11]	Measures symptom severity and interference with daily life	Symptom severity (e.g., pain, fatigue, nausea, shortness of breath) Symptom interference (e.g., mood, work, relations, enjoyment of life)	Cancer patients in clinical and research settings
PROMIS [12]	Provides standardized assessment of physical, mental, and social health	Physical health (pain, fatigue, physical function) Mental health (anxiety, depression) Social health (participation, support)	General population and disease-specific cohorts
EORTC QLQ-C30 [13]	Assess cancer-specific health-related quality of life	Global health status Physical, role, emotional, cognitive, social functioning Symptom scales (e.g., fatigue, nausea, pain)	Cancer patients in clinical trials worldwide
EQ-5D [14]	Provides a simple, generic measure of health status for clinical and economic evaluation	Mobility Self-care Usual activities Pain/discomfort Anxiety/depression Visual Analog Scale for overall health	General population and patients with chronic diseases, including cancer

Abbreviations: EORTC QLQ-C30, European Organisation for Research and Treatment of Cancer Quality of Life Questionnaire; EQ-5D, EuroQol 5-Dimension instrument; FACT, Functional Assessment of Cancer Therapy; HRQL, health-related quality of life; MDASI, MD Anderson Symptom Inventory; PROMIS, Patient-Reported Outcomes Measurement Information System.

**Table 2:** Health-Related Quality-of-Life (HRQL) Outcomes with Immune Checkpoint Inhibitors.

Drug/Regimen (*n* *)	Checkpoint Target	Cancer Type(s)	Study/Trial	HRQL Instrument(s)	Key HRQL Findings	PRO Endpoint Status
Nivolumab (*n* = 292)	PD-1	NSCLC	CheckMate 057 [9]	EQ-5D, LCSS	Better lung-symptom control and global health status vs. docetaxel; delayed HRQL deterioration	Secondary endpoint
Pembrolizumab (*n* = 154)	PD-1	NSCLC	KEYNOTE-024 [4]	EORTC QLQ-C30	Improved or stable HRQL at 3–4 months; slower decline in cough, pain, dyspnea vs. chemotherapy	Secondary endpoint
Pembrolizumab (*n* = 556)	PD-1	Melanoma	KEYNOTE-006 [20]	EORTC QLQ-C30	Higher global HRQL and functioning vs. ipilimumab	Secondary endpoint
Nivolumab (*n* = 410)	PD-1	Renal cell carcinoma	CheckMate 025 [19]	FACT-FKSI-DRS	Greater improvement in kidney symptom index and faster HRQL gain vs. everolimus	Secondary endpoint
Atezolizumab + Chemotherapy (*n* = 201)	PD-L1	Small cell lung cancer	IMpower133 [21]	EORTC QLQ-C30, -LCSS	Sustained HRQL improvement vs. chemotherapy alone; prolonged symptom relief	Secondary endpoint
Durvalumab (*n* = 476)	PD-L1	Stage III NSCLC (post-CRT)	PACIFIC [22]	EORTC QLQ-C30 & -LC13	Stable HRQL vs. placebo; no worsening in cough, dyspnea, fatigue	Secondary endpoint
Avelumab (BSC maintenance) (*n* = 350)	PD-L1	Urothelial carcinoma	JAVELIN Bladder 100 [24]	FACT-Bladder, EQ-5D	No significant HRQL decline; similar time to deterioration vs. BSC alone	Secondary endpoint
Nivolumab + Ipilimumab (*n* = 550)	PD-1 + CTLA-4	Renal cell carcinoma	CheckMate 214 [25]	FKSI-19	Higher HRQL scores vs. sunitinib; better symptom and functional outcomes throughout follow-up	Secondary endpoint
Nivolumab ± Ipilimumab (*n* = 314)	PD-1 ± CTLA-4	Melanoma	CheckMate 067 [6]	EQ-5D, EORTC QLQ-C30	Stable global HRQL despite more toxicity; rebound after discontinuation	Secondary endpoint
Durvalumab + Tremelimumab (STRIDE) (*n* = 393)	PD-L1 + CTLA-4	Hepatocellular carcinoma	HIMALAYA [26]	EORTC QLQ-C30 & -HCC18	Slower HRQL decline vs. sorafenib; similar HRQL between durvalumab alone and STRIDE combination	Secondary endpoint
Pembrolizumab (*n* = 296)	PD-1	Gastric/GEJ cancer	KEYNOTE-061 [28]	EORTC QLQ-C30	HRQL preserved vs. chemotherapy despite modest response rate	Secondary endpoint
Multiple ICIs (real-world) (*n* = 93)	PD-1/PD-L1 ± CTLA-4	Mixed cancers	Alwhaibi 2025 [29]	EQ-5D	Immunotherapy users reported better overall HRQL than chemotherapy users in practice	N/A (observational HRQL outcome)

* *n* reflects the number of randomized patients in the immune checkpoint inhibitor arm (intention-to-treat population). For the real-world observational cohort (Alwhaibi 2025 [29]), *n* reflects the HRQL-evaluable survey sample. Abbreviations: BSC, best supportive care; CRT, chemoradiotherapy; CTLA-4, cytotoxic T lymphocyte–associated antigen 4; GEJ, gastroesophageal junction; FKSI-19, revised FACT Kidney Symptom Index; FKSI-DRS, FACT Kidney Symptom Index–Disease-related Symptoms; ICI, immune checkpoint inhibitor; LC13, lung cancer supplement to EORTC QLQ; LCSS, lung cancer symptom scale; NSCLC, non–small cell lung cancer; PD-1, programmed cell death protein 1; PD-L1, programmed death ligand 1.
